# Buffering properties of fish aquafeeds: concepts, determinants and effects on physiology and growth

**DOI:** 10.3389/fvets.2026.1919609

**Published:** 2026-09-17

**Authors:** Lorenzo Márquez, Adrián J. Hernández, Rosendo L. Azcuy, Nancy E. Ruiz, Paola Orellana, Majorie Larson, Gabriel A. Morales, Manuel Díaz, Francisco J. Moyano, Eduardo Almansa

**Affiliations:** 1 Centro de Investigación, Innovación y Creación, Vicerrectoría de Investigación y Posgrado, Universidad Católica de Temuco, Temuco, Chile; 2 Laboratorio de Nutrición y Fisiología de Peces, Depto. de Ciencias Agropecuarias y Acuícolas, Facultad de Recursos Naturales, Universidad Católica de Temuco, Temuco, Chile; 3 Universidad de Buenos Aires, Facultad de Agronomía, Departamento de Producción Animal, Buenos Aires, Argentina; 4 Escuela de Medicina Veterinaria, Facultad de Recursos Naturales y Medicina Veterinaria, Universidad Santo Tomás, Temuco, Chile; 5 Instituto de Investigaciones en Producción Animal, Consejo Nacional de Investigaciones Científicas y Técnicas INPA–CONICET, Buenos Aires, Argentina; 6 Departamento de Biología y Geología, Facultad de Ciencias Experimentales, Universidad de Almería, Almería, Spain; 7 Centro Oceanográfico de Canarias, Instituto Español de Oceanografía, (COC-IEO), CSIC, Santa Cruz de Tenerife, Spain

**Keywords:** acid binding capacity, aquafeeds, digestive physiology, fish growth, intestinal microbiota, specific dynamic action

## Abstract

The present paper is a comprehensive review of the existing literature on aquafeed acid binding capacity (ABC), and its effects on the digestive physiology, metabolism, intestinal microbiota, and growth of farmed fishes. ABC can be defined as the quantity of acid (usually HCl) required to reduce diet pH to a specified endpoint, typically 3.0 (ABC-3) or 4.0 (ABC-4). The ABC of a diet may influence the secretion pattern and minimum gastric pH, as well as the total amount of HCl secreted during digestion. On the other hand, the acidity of the gastric lumen plays a crucial role in the digestive processes of many cultured fish species, facilitating the activation of gastric proteases, the initial hydrolysis of dietary proteins, and the solubilization of mineral nutrients. As the secretion of gastric HCl entails energy expenditure, it is plausible that the fraction of the specific dynamic action (SDA, defined as the energetic costs due to food digestion and assimilation), associated with gastric digestion in fish could be modulated through dietary formulations and their corresponding ABC. In fish, available information suggests that a decrease in diet ABC can lead to a reduction in total specific dynamic action (SDA), while the effects on growth rates remain ambiguous or transitory. Modifications to diet ABC through supplementation with calcium carbonate (CaCO₃) may negatively impact growth rates due to a concomitant decrease in phosphorus digestibility rather than an increase in HCl secretion. Furthermore, when the costs of HCl secretion were examined using proton-pump inhibitors, such as omeprazole, both SDA values and durations decreased similarly, being this reduction directly correlated with diminished fish growth rates. Regarding the influence of dietary buffering properties on fish gut microbiota, from the few available studies it can be deduced that the effects are not very significative, but this could be just a consequence of the immaturity of the research on diet ABC and fish microbiota. Further research is warranted to determine whether the manipulation of dietary ABC can lead to enhanced growth rates and improved intestinal health in cultured fish.

## 1 Introduction

The digestive function in fish is a result of the intricate interplay between the physiological capacity of the organism to regulate conditions within the digestive tract, the activity of digestive microbiota, environmental factors such as temperature, and physicochemical properties of ingested feed. Among the various factors that can potentially influence the digestive process is the dietary acid binding capacity (ABC). However, the effects of diet ABC on digestion have not been comprehensively reviewed in the case of fish aquafeeds.

The progressive replacement of fish meal by plant-derived ingredients in commercial aquafeeds (1–3) can have impacted aquafeed ABC. This nutritional transition has modified the chemical composition, and physicochemical properties of fish aquafeeds. It is well known that a low gastric pH promotes the activation of pepsinogens (the zymogen secreted by the stomach), the activity of pepsins (the main gastric proteolytic enzymes), the solubilization of some minerals such as ferric iron and calcium, and it also reduces the microbial load contained in the ingested food in vertebrates (4–7). Thus, since the pH-buffering properties of aquafeeds can affect the gastric pH of fish, it is inferred that those properties can also interfere with the aforementioned gastric processes and, hence, with digestive efficiency and nutrient utilization.

Dietary ABC influences the resistance of the feed to acidification within the stomach, potentially changing gastric pH. This, in turn, can affect key gastric functions, including protein hydrolysis, microbial load reduction, and the absorption of certain minerals like ferric iron or calcium of dietary origin (5). For instance, high-ABC diets in terrestrial vertebrates are associated with elevated gastric pH, which can facilitate the proliferation of pathogenic microorganisms and increase the risk of intestinal diseases (8). In addition, diet ABC may also affect the energetic costs associated with digestion, although it is not clear if the secretion of gastric acid makes a significant contribution to these costs. For instance, Ferreira et al. (9) compared a knock-out strain of the fish *Astyanax mexicanus* lacking a functional gastric proton pump with a wild type strain, and reported a 22% reduction in SDA in the knock-out strain. However, the same authors calculated that the direct cost of translocating protons into the stomach of this species during digestion only amounted to 2–6% of the total SDA.

Organic acids are used as acidifiers for animal diets (10) and can change diet ABC by reducing the pH of the diet before being ingested, but they are only one factor among the many ones affecting it. The overall ABC of a diet is also influenced, at least, by ingredient composition, protein sources, mineral content, and the inclusion of acidifying or buffering compounds (11, 12). Therefore, studies focusing solely on dietary acidifiers cannot fully explain the physiological implications of dietary ABC in fish.

Emerging research on fish physiology suggests that diet ABC may influence gut pH, modulate the intestinal microbiota, and even impact growth rates in aquaculture species (12–14). Given the growing body of literature on the effects of dietary ABC on fish, this work aims to comprehensively review the topics related to the buffering properties of fish diets: factors affecting the ABC of aquafeeds for fish, the range and variability of ABC in experimental and commercial aquafeeds, and the reported effects on fish physiology. Several theoretical and experimental issues have been considered particularly relevant: (i) the role of organic acids as diet acidifiers, and the effects on buffering properties of fish diets, (ii) the integration of published data about factors influencing gut pH, and data on the buffering capacity of aquafeeds, (iii) potential difficulties when applying the concept of ABC to intestinal physiology, where alkalization processes prevail, (iv) temporal patterns of ABC of commercial aquafeeds, (v) methodological interferences that may arise when using phosphate salts to manipulate diet ABC. All these topics are of importance to understand the consequences of diet buffering properties on current fish aquaculture.

## 2 Bibliography search, data tabulation, and statistics

Bibliography selection was based on the results obtained from Google Scholar after inserting the terms “acid binding capacity” or “buffering capacity” and “diet,” and also the same terms in combination with “aquaculture,” “fish,” “poultry” or “pig.” Literature was searched during August–September 2024, and again during April–May 2026. The results were ordered according to content relevance, firstly without a defined period of time, and again during the last 5 years. Research articles, review articles and book chapters from internationally recognized journals and publishing houses, and also reports from recognized research institutions were all considered as suitable scientific works. Output articles were firstly checked according to titles. Abstracts were skimmed for relevant concepts, and contents scanned for specific measurements of diet pH, and ABC (or buffering capacity). Data on diet formulation, inclusion of buffering or acidifying additives, diet pH, and ABC until pH 3.0, 3.5, and 4.0 (ABC-3, ABC-3.5, ABC-4) were tabulated. Data on diet pH were classified according to the presence/absence of dietary additives (diet + acidifiers, diet without additives, diet + CaCO_3_), and the mean and SD of diet pH were calculated for each dietary group. pH data were analyzed by means of a robust Anova (Jamovi software, Module Walrus, method based on trimmed means with trim level = 0.2). Potential correlations between diet ABC-3 and the inclusion of fishmeal or plant protein sources were explored by means of the Spearman correlation coefficient, setting the significance level at *p* = 0.05.

For the specific section about formulation patterns of commercial diets for Norwegian salmon, the most recently published articles (2, 15) were checked for earlier works, including technical reports released by Nofima (16–19). Based on the principle of ABC additivity, and published values of ABC until pH 4.0 (ABC-4), and ABC until pH 3.0 (ABC-3) for feed ingredients (11, 20), temporal changes in ABC of commercial diets for Norwegian salmon was estimated (Supplementary material). For this estimation, we assumed that ABC values of a few unexamined ingredients were comparable to those of similar ingredients. The ABC-4 of soybean protein concentrates (SPC) was taken from the work by Stas et al. (20). However, as the ABC-3 of SPC was not measured in that study, we estimated that value according to: (i) ABC-3 of soybean meal [1,068 mEq H^+^ kg^−1^ according to Lawlor et al. (11)], and (ii) the ratio of SPC ABC-4 to soybean meal ABC-4:

$$\frac{\mathrm{ABC}­4(\mathrm{SPC})\;\text{according to Stas}\;\mathrm{et}\;\mathrm{al}.(20)}{\mathrm{ABC}­4(\text{soybean meal})\;\text{according to Stas}\;\mathrm{et}\;\mathrm{al}.(20)}=\frac{737}{602}$$

As a result, ABC-3 of soybean meal (11), and ABC-3 of SPC (estimated) were set at 1068 and [(737/602) × 1,068] = 1,308 mEq H^+^ kg^−1^, respectively. Potential correlations between time (year) and ABC-4 or ABC-4 of commercial diets were explored by means of the Spearman correlation coefficient.

For the section on the physiology and regulation of gut pH in fish, the terms “gastric acid secretion”, “gut pH” or “alkaline tide” and “fish” were also used. Comparisons with non-fish species were mainly based on recent and relevant reviews on terrestrial monogastric vertebrates, but the comparison with snakes was more extensively discussed due to the similarity between the research questions studied in snake-based and fish-based articles. In addition to Google Scholar, personal research experience of authors on diet additives, diet pH and gut physiology in fish was also important to complete the list of references.

## 3 Buffering properties of aquafeeds: measurement and determinants in fish aquafeeds

### 3.1 Diet acid binding capacity

The ABC of a nutritional matrix is defined as the amount of hydrogen ions (H^+^) required to reduce the pH of a unit mass suspended in water from its initial value to a specific endpoint, typically 4.0 (ABC-4) or 3.0 (ABC-3) for fish. ABC is commonly expressed in mmol H^+^ kg^−1^ (mEq H^+^ kg^−1^), or μmol H^+^ g^−1^ (μEq H^+^ g^−1^). Closely related to ABC is the concept of buffering capacity (BC), which is defined as that amount of H^+^ per unit decrease in pH, mathematically expressed as BC = ABC/(−ΔpH) (11). Therefore, BC can be understood as the normalization of ABC with respect to the unit of pH. For a fixed titration endpoint, BC has been used as an additional variable for the comparison and selection of dietary ingredients for the aquafeeds for a given fish species, since BC was assumed to affect pepsin activity (21).

ABC should not be considered an intrinsic property of a single nutrient or ingredient but rather an integrated physicochemical characteristic resulting from the interaction among multiple dietary components and their respective dissociation constants (pKa values) (11, 20, 22).

A useful tool for evaluating the buffering properties of a particular ingredient or aquafeed is plotting the HCl titration curve, which represents the relationship between pH and the cumulative addition of H^+^ ions (7, 23–25). Titration curves provide information regarding the magnitude and distribution of buffering reactions across different pH ranges, allowing the identification of buffering regions associated with specific chemical constituents of the feed matrix. Therefore, titration curves may provide a more comprehensive characterization of dietary buffering properties than endpoint measurements alone.

### 3.2 Measurement protocol and determinants of acid binding capacity in fish diets

The method for measuring diet ABC typically involves stirring an aqueous suspension of the milled diet or ingredient until the initial pH stabilizes (11). A small aliquot of a strong acid, such as hydrochloric acid (HCl), is then added, and after the pH stabilizes, the process is repeated with successive acid additions until the target pH (usually 4.0 or 3.0) is reached. Despite its widespread use, methodological variations among studies include differences at least in: (i) particle size [2 mm in Lawlor et al. (11), 0.4 mm in Stas et al. (20), generally not reported in articles on aquafeeds], (ii) diet-to-water ratio [1:100 in Ciavoni et al. (14), 1:10 in Bravo et al. (24), 1:5 in Márquez et al. (7) and Bertini et al. (26)], (iii) endpoint pH [3.0 in Parma et al. (13) and Monge et al. (27), whole titration curve in Márquez et al. (7, 23) and Bravo et al. (24)], and (iv) stabilization time [60 s in Bravo et al. (24), 30 s in Márquez et al. (7, 23) and Monge et al. (27)]. This variability highlights the necessity for a standardized protocol when measuring the initial pH and ABC of aquafeeds, as emphasized previously in studies about swine nutrition (22). However, a completely standardized measurement protocol will be pointless. A consensus among researchers that takes into account the peculiarities of each species will be much more useful. For instance, the titration endpoint should be related to the minimal gastric pH found in each species. Indeed, the publication of whole titration curves for aquafeed ingredients till a low enough pH (e.g., 2.0) will make it possible the estimation of diet ABC (see section 3.3) for a wide range of fish species. Until such a consensus is reached, a preliminary standard protocol can be proposed as follows. Since the moisture content of gastric chyme varies between 50 and 80% in several cultured fish species (24, 28, 29), a sound diet-to-water ratio will be 1:5. In this way, the water content will be high enough to allow an efficient stirring of the mix during titration, and it will be close to the range of moistures of the gastric chyme. As for particle size, this parameter is usually not reported in articles showing data on aquafeed ABC, but in the case of diets for livestock, 2 mm and 0.4 mm has been used (11, 20). We proposed a particle size of 0.4 mm or smaller since, on the one hand, 2 mm is too large for the pellets supplied to juvenile fish which can have a diameter of 2 mm and, on the other hand, the current literature already contains a list of ingredient ABCs based on a particle size of 0.4 mm. In relation to the stabilization time, we proposed the time reported in the majority of articles showing data on aquafeed ABC, i.e., 30 s. As for the titration endpoint, it should be selected according to the gastric physiology of the studied species. For example, in juvenile Atlantic salmon we suggest an endpoint of 3.5 (24). In juvenile rainbow trout, an endpoint of 4.25 seems appropriate (29–31). In juvenile gilthead sea bream, we proposed a titration endpoint of 4.5 (7, 32). In the case of Nile tilapia, the highly acidic gastric chyme suggests an endpoint as low as 1.5 (25).

#### 3.2.1 Stabilization time

The time required for pH stabilization after each acid addition varies depending on the food matrix. Insufficient stabilization time could result in inaccurate estimation of ABC values and reduce the comparability among studies. After acid addition, several physicochemical processes occur simultaneously, including dissolution of feed particles, diffusion of hydrogen ions into the matrix, ion exchange reactions, and protonation of weak acids and bases. These processes may proceed at different rates depending on ingredient composition and physical structure. This delay may be attributed to the unique buffering compounds present in different protein sources.

#### 3.2.2 Particle size

Since buffering compounds dissolve and proton exchange occurs at the surface of food particles, there is often a negative relationship between particle size and ABC values. As particle size decreases, the specific surface area available for dissolution and proton exchange increases, facilitating the release of buffering compounds into solution and accelerating acid–base reactions. Furthermore, grinding may disrupt cellular structures and expose previously inaccessible chemical groups capable of participating in buffering reactions.

Studies on commercial limestones (sometimes used as a calcium source) have demonstrated such a relationship, though the correlation was weak and statistically non-significant (33). While some authors standardize particle size in their ABC measurements (11, 20), no systematic investigations have been conducted to assess the specific effects of particle size on the ABC of aquafeed ingredients or finished feeds. Future investigations should address this knowledge gap, particularly considering that grinding intensity and particle size distribution may vary among feed manufacturing processes.

#### 3.2.3 Buffering effect of proteins

Ionizable groups in the food matrix, with a pKa between the initial pH and the titration endpoint, can counteract the effects of added H^+^. In aquafeeds, proteins and peptides exert significant buffering effects in the stomach due to the carboxylic groups in the side chains of aspartic acid (Asp) and glutamic acid (Glu), which have pKa values between 2.0–4.0 and 3.0–4.0, respectively. The imidazole group in histidine (His) becomes semi-ionized between pH 5.0–7.0 (34). Consequently, proteinaceous ingredients, particularly fishmeal, soybean meal, and soybean protein concentrates, exhibit the highest ABC among organic ingredients (11, 20). Since Asp and Glu are more abundant than His in proteins, their buffering effects are the most pronounced within the pH range of 4.5–3.5 (midway through fish digestion), rather than at higher pH values (6.5–5.0).

#### 3.2.4 Supplementation with crystalline amino acids

Methionine (Met) and lysine (Lys) are the most commonly supplemented amino acids in fish diets, particularly in plant-based aquafeeds (35). The α-carboxylic groups of Met and Lys exhibit pKa values near 2.2 at 25 °C and an ionic strength of 10 mM (36), contributing minimally to diet ABC-4 but potentially influencing ABC-3, as noted by Lawlor et al. (11). However, given that the gastric pH in most fish species generally remains above 3.0 during digestion, their impact is limited. Taurine, another important crystalline amino acid added to plant-based fish diets (37), has pKa values around 1.5 and 8.8, respectively (38). Thus, taurine likely does not act as a buffering agent in the stomach of fish.

#### 3.2.5 Phosphate ions

Phosphate ions are unlikely to have a large impact on diet ABC-4 or ABC-3, as only one of the three pKa constants (pKa₂ = 7.2 at 25 °C) falls near, but still above, the typical pH of fish diets (39). Moreover, mineral phosphates derived from fish bone (present in fishmeal) are mainly in the form of hydroxyapatite and tricalcium phosphate (40), both of which have very low solubility (41). However, commercial fish diets may be supplemented with more soluble phosphate salts, such as monosodium phosphate and monocalcium phosphate, which are more soluble at acidic pH (42). Additionally, the use of commercial phytases in plant-based aquafeeds has been shown to increase the concentration of soluble phosphate in the stomach (pH 6.2–4.5) and proximal intestine (pH 6.4–6.6) of juvenile *Sparus aurata* (32). In any case, solubilized phosphates can effectively buffer intestinal contents at near-neutral pH.

#### 3.2.6 Plant vs. animal ingredients

According to Lawlor et al. (11), plant-based protein ingredients, particularly whole cereals, generally have lower ABC values than animal-derived ingredients. For example, wheat has an ABC-4 of approximately 110 mEq kg^−1^, while lupins, sunflower meal, and soybean meal have ABC-4 values close to 340, 480, and 640 mEq kg^−1^, respectively. In contrast, meat and bone meal and fishmeal exhibit ABC-4 values of approximately 600 and 740 mEq kg^−1^, respectively. This distinction is particularly relevant in commercial fish diets, where the inclusion of fish protein ingredients has decreased from 64 to 12%, while the proportion of plant protein ingredients has risen from 0% to over 40% in the past 30 years (Supplementary material).

#### 3.2.7 Data about ABC and diet pH in experimental aquafeeds

Table 1 presents data on the pH and buffering properties of experimental aquafeeds measured according to the protocol discussed previously. The experimental diets exhibit an ABC-4 ranging from 214 to 730 mEq kg^−1^, with ABC-3 values between 212 and 1,200 mEq kg^−1^. As expected, in those works assaying two or more titration endpoints, the lower the endpoint the higher the measured ABC for a given diet. The initial pH of experimental diets tends to be mildly acidic (6.0 ± 0.3) in the absence of acidifiers or buffers, whereas diets including strong or organic acids showed lower pH (5.0 ± 0.8), and the pH of diets with CaCO_3_ was higher (6.3 ± 0.2) (Table 2). A robust Anova (Jamovi software, Module Walrus) resulted in a significant difference in pH among dietary groups (*p* = 0.034). Equilibration time was usually 30 s, but Bravo et al. (24) set it at 60 s, and Ciavoni et al. (14) used continuous titration with a pH-stat device. It is interesting to note that continuous titration produced particularly low ABC values. However, the range of equilibration times assayed is too limited to conduct a regression line. The same limitation applies to the diet-to-water ratios, whereas no data on the particle size of the titrated diet was supplied by researchers studying aquafeeds.

**Table 1:** Composition, pH, and buffering properties of experimental aquafeeds for fish.

Reference	FM/Anim. protein	Plant/SCP protein	CP	Acid/buffer	Diet pH	ABC 4.0	ABC 3.5	ABC 3.0
Sugiura et al. (6)	25	43	37	0	5.70			
	24	41	35	5% HCl	4.00			
	24	41	36	3.5% H_2_SO_4_	3.70			
	25	43	37	1% H_2_SO_4_	4.80			
	24	41	35	5% CH_3_COOH	4.70			
	24	41	35	5% CaCO_3_	6.00			
Márquez et al. (7)	>30	>20	42	0	6.00	470		
Márquez et al. (23)	63	20	48	0	6.15	670	1,090	
	63	20	48	0	6.05	710	1,130	
	63	20	48	1.1% NDF	5.85	730	1,170	
	63	20	48	1.1% NDF	5.85	670	1,090	
	35	47	44	0	6.05	370	610	
	35	47	44	0	6.05	430	690	
	35	47	43	1.1% NDF	5.55	410	730	
	35	47	43	1.1% NDF	5.55	450	770	
Monge et al. (27)	59	26	45	0	6.10			1,200
	20	53	45	0	6.20			900
	5	64	45	0	6.60			800
Bravo et al. (24)	nd	nd	43	0	6.03	300		700
Parma et al. (13)	20	68	47	0	6.40			750
	10	77	47	0	6.40			530
	10	74.5	47	3% CaCO_3_	6.50			750
Ciavoni et al. (14)	2	56	36	0	5.96			212
	1.9	54	35	3% CaCO_3_	6.36			328
	2	72	57.5	0	5.75			284
	1.9	70	55.5	3% CaCO_3_	6.43			406
Bertini et al. (26)	3	70	32	0	6.01	316	475	652
	3	69	31	0	5.63	272	424	592
	3	75	31	0	5.23	214	346	534

FM/Anim. protein: fishmeal + animal protein ingredients. Plant/SCP protein: plant plus single cell protein ingredients. CP, crude protein. ABC 4.0 and ABC 3.5: Acid binding capacity to pH 4.0 and 3.5, respectively. NDF: Sodium diformate. Nutrients are given in % of dry matter.

**Table 2:** Mean and standard deviations of pH values of experimental aquafeeds presented in Table 1, and analysis with a robust ANOVA (Jamovi software).

Aquafeed group	*n*	Mean pH	SD	*F*	*p*-value
With acidifier	8	3.70	0.83	5.74	0.034
Without additives	17	5.23	0.32		
With CaCO_3_	4	6.00	0.22		

Diets were grouped according to the presence or absence of acidifying/buffering additives.

In relation to diet formulation, the contents of fishmeal plus animal protein ingredients in diets presented in Table 1 varied from 0 to 60%, whereas the inclusion of plant plus single-cell ingredients approximately ranged from 25 to 80%. The ABC-3 of experimental diets showed a positive correlation with the inclusion rate of fishmeal and animal protein ingredients (Spearman’s rho = 0.717, *n* = 13, *p* = 0.006), while the correlation with plant-based and single-cell protein ingredients was negative (Spearman’s rho = −0.858, *n* = 13, *p* < 0.001). The significance of such correlations indicates a good consistency between expectations and experimental measurements.

### 3.3 Additivity of ingredient ABCs and factors influencing aquafeed ABC

The relationship between an ingredient’s nutrient composition and its ABC is complex, but some studies have reported positive correlations with protein and ash content (43). When examining whole diets composed of multiple ingredients, experimental evidence supports the additivity of ingredient ABCs. This means that diet ABC can be accurately estimated as the weighted average of the ABCs of the individual ingredients, based on their inclusion rates. Jasaitis et al. (43) demonstrated this concept by formulating two isonitrogenous diets for cattle using four different ingredients with known buffering capacities, and predicted values closely matched measured ABCs. Similarly, Lu et al. (44) formulated six diets for pigs with varying ingredient compositions (2–7 ingredients) and observed ABC-3 values between 800 and 900 mEq kg^−1^, which correlated well with the predicted ABC values. These studies provide strong evidence supporting the validity of ABC predictions based on the additivity of ingredient ABCs. Consequently, a general strategy to formulate aquafeeds while taking into account diet ABC would consist in applying the additivity principle to estimate the ABC of a given dietary formula. All the ingredients will make a fractional contribution to diet ABC. Considering the impact of macro-ingredients (for instance protein sources), or the presence of minor ingredients with a high ABC (e.g., CaCO_3_) on the final ABC, can be as useful as considering the presence of acidifiers. Of course, diet ABC would be only one of the variables to be considered when formulating an aquafeed. Most probably other variables, such as the quantity and quality of protein, will be more important to formulators than ABC, but considering ABC just as an additional constraint within a hierarchical list of nutritional constraints will be useful. Nevertheless, when the formulator cannot select macro-ingredients with a low ABC (due to economic, nutritional or availability constraints), the most practical step to reduce diet ABC continues to be the inclusion of an acidifier.

Several factors may influence the accuracy of predicted ABC values, leading to discrepancies between predicted and measured ABCs. One factor involves interactions between ingredients, particularly the effect of phytate on soluble proteins. Plant ingredients used in fish diets often contain phytate at concentrations ranging from 0.15 to 3% (45). Phytate interacts with soluble proteins, either directly or via divalent cations, forming insoluble macromolecular complexes, especially in the acidic environment of the stomach—a phenomenon also observed in fish species (46, 47). The precipitation of soluble proteins in the gastric chyme could reduce the ABC of gastric digesta, although this effect remains largely unexplored. Nevertheless, this interaction likely affects a small fraction of dietary proteins, as only a portion of these proteins exists in a soluble form. For instance, a phytate-containing diet reduced the soluble protein content in the stomach of *Sparus aurata* from 3 mg to 2 mg of soluble protein per gram of dry matter, despite the fish being fed a diet containing 42% crude protein (32). Therefore, the overall impact of protein precipitation on the total dietary protein content is small.

Another factor potentially influencing the prediction of diet ABC is the initial pH of an aqueous suspension of aquafeed, which typically ranges from 6.6 to 5.5 (Table 1). In contrast, the initial pH of the titration solutions for some ingredients can deviate significantly from this range (11). For example, ingredients with initial pH values outside the 6.6–5.5 range include: (i) organic acids (pH 1.6–3.5), (ii) salts of organic acids (pH 7.4–8.6), (iii) metallic oxides (pH 8.3–8.8), and (iv) bicarbonates and carbonates (pH 8.7–8.9). As a result, the ABC of these compounds, when exposed to gastric chyme, will differ from the values observed in water suspensions. Nevertheless, these ingredients are typically included at low levels, and organic acids or carbonates are not consistently present in fish diets.

Another consideration regarding organic acids is their overlooked buffering capacity. Organic acids are weak acids containing ionizable groups, primarily carboxylic groups (-COOH), with pKa values between 3.0 and 6.0 (10). When added in their protonated form, these acids reduce the pH of fish diets (48). However, if they are only added as a salt (e.g., potassium citrate, sodium citrate, calcium formate), their effect is the opposite (11). Even when added in their protonated form, organic acids act as buffers near their pKa, resisting pH changes. As HCl secretion lowers gastric pH below 4.0, the buffering effect of organic acids becomes more pronounced. Consequently, the ranking of ABC values for acidified diets can depend on the titration endpoint used in the protocol. For instance, Márquez et al. (23) investigated the impact of sodium diformate (NDF)—a commercial blend of sodium formate and formic acid—on two sets of fish diets based on fishmeal (FM) and barley protein concentrate (BPC), respectively. NDF reduced ABC-5.0 and lowered the initial pH by 0.3–0.5 units in both diet groups. However, ABC-4.0 remained unaffected by NDF and, in the case of BPC-based diets, NDF slightly increased ABC-3.5. This suggests that evaluating the effects of organic acidifiers, particularly in relation to HCl sparing, requires consideration of the species-specific minimal gastric pH. Additionally, the effects of organic acidifiers and their salts may not be solely due to their acidifying or buffering properties. Some compounds can be absorbed by enterocytes and utilized as cellular energy sources (49), or they may influence intestinal microbiota (50). Nonetheless, acidifying additives are not commonly included in either experimental or commercial fish diets, and when they are used, the inclusion rates are generally below 2% (50, 51).

An additional consideration that does not affect the additivity of ABC in the original diet is the variation in ABC of stomach contents throughout the digestion process. The ABC of gastric chyme can diverge from the ABC of the diet, particularly towards the end of gastric digestion. This divergence arises because the concentration of ionizable groups in the gastric contents is influenced not only by the initial ionizable groups present in the diet, but also by the new ionizable groups generated through nutrient hydrolysis (52). For instance, the cleavage of a single peptide bond results in the formation of one carboxylic group with a pKa value between 3.0 and 4.0, alongside one amine group with a pKa in the range of 8.0 to 9.0. The pH of fish gastric contents typically fluctuates between 3.0 and 5.0 (7, 13, 24, 29, 53). Consequently, during gastric digestion, newly formed amino groups will acquire a proton, while newly generated carboxylic groups will remain partially ionized towards the end of the digestion process. Each broken peptide bond thus removes less than one proton from the liquid phase of the gastric chyme when the pH is optimal for fish gastric proteases. This process has been shown to lead to a modest increase in ABC; for example, *in vitro* digestions of whey protein with porcine pepsin resulted in a 16% increase in ABC (52). Additionally, during *in vitro* digestion of defatted soybean meal (containing over 60% protein) with gastric extracts from rainbow trout at 15 °C and pH 3.5, the proportion of proteins measured as 0.3–1.7 kDa oligopeptides increased from 15 to 45% (4). This suggests that the phenomenon of increasing ABC in the digesta is likely applicable in fish as well. However, the findings of Mat et al. (52) indicate that the increase in ABC during gastric digestion constitutes only a small fraction of the original diet ABC, and diet ABC is easier to measure, and simpler to predict than ABC of digesta.

In summary, all the factors discussed appear to exert small interferences with the principle of ABC additivity, with their combined effects potentially yielding measurable impacts in specific instances. In the particular case of experimental aquafeeds containing unusual high contents of phytate, organic acids and/or certain minerals, the additivity principle could perhaps be challenged. However, as demonstrated by the experiments conducted by Jasaitis et al. (43) and Lu et al. (44), the empirical correlation between measured ABC and predicted ABC remains robust in multi-ingredient diets such as current fish aquafeeds.

### 3.4 The case of aquafeeds for Norwegian farmed salmonids: formulation and implications for diet ABC

Data from the relevant literature indicate a steady increase in the inclusion of plant-based ingredients in commercial diets for Norwegian salmon, accompanied by a concomitant decrease in the inclusion of fish meal (2). Based on the empirical measurements of ABC-3 and ABC-4 of dietary ingredients (11), the estimation of ABC-3 of soybean protein concentrate (as explained in section 2), and published data about the ingredients used in the production of salmonid aquafeeds (2, 3), we have estimated the change in ABC of diets supplied to Norwegian salmonids during the years 1990, 2000, 2010, 2012, 2013, 2016, and 2020. The average estimated values of ABC-4 and ABC-3 for salmon diets during the period from 2010 to 2020 were 460 mEq H^+^ kg^−1^ and 880 mEq H^+^ kg^−1^, respectively. These values fall within the ABC ranges reported for experimental fish diets (2012–2023), which are 210–730 mEq H^+^ kg^−1^ for ABC-4, and 210–1,200 mEq H^+^ kg^−1^ for ABC-3 (Table 1).

The estimates for ABC-4 and ABC-3 in commercial diets for Norwegian salmon decreased by approximately 20% from 1990 to 2020 (Table 3, Supplementary material), coinciding with an increase in the inclusion rate of plant protein ingredients from 0% to over 40%. The most significant decline in ABC occurred between 1990 and 2010, a period marked by a substantial reduction in fishmeal inclusion. In contrast, estimates for ABC during the period from 2010 to 2020 exhibited only minor fluctuations, attributed to variations in the inclusion rate of soybean protein concentrate (SPC), which is the plant protein source with the highest ABC. The correlations between time (year) and ABC-4 (Spearman rho = −0.821, *n* = 7, *p* = 0.034), or ABC-3 (Spearman rho = −0.821, *n* = 7, *p* = 0.034) were negative. In summary, salmon diets have been gradually reducing their ABC over the past few decades, largely due to the progressive replacement of marine ingredients with vegetable sources. However, these temporal trends should be interpreted with caution given the limited sample size (*n* = 7). In the coming years, additional estimations, and direct measurements of ABC of salmon aquafeeds produced in Norway will be necessary to confirm the trends herein reported.

**Table 3:** Estimations of mean ABC-3 and ABC-4 of aquafeeds produced in Norway for farmed salmon during the last 30 years, based on literature data.

Year	ABC-3 (mEq H^+^/kg)	ABC-4 (mEq H^+^/kg)	Plant ingredients (%)	Reference
1990	1,110	570	0.00	Sørensen et al. (16)
2000	1,019 (964)	543 (505)	18.8	Sørensen et al. (16)
2010	889	459	35.4	Ytrestøyl et al. (17)
2012	891	468	37.9	Ytrestøyl et al. (19)
2013	901	478	36.5	Ytrestøyl et al. (18)
2016	852	440	40.2	Aas et al. (15)
2020	873	457	46.6	Aas et al. (2)

ABC estimations for diets produced during the year 2000 were calculated according to two scenarios: (i) plant protein sources only consisted of soybean protein concentrate, and (ii) plant protein sources followed the same ingredient distribution reported for diets produced in 2010 (figures in parenthesis).

More recently, Aas et al. (3) provided data on the ingredients used to formulate commercial diets for Norwegian rainbow trout in 2020. Using the same methodology applied to Norwegian salmon diets, the estimated ABC-4 and ABC-3 for commercial diets for Norwegian rainbow trout in 2020 were found to be at least 452 mEq H^+^ kg^−1^ and 847 mEq H^+^ kg^−1^, respectively.

## 4 Physiological principles determining gut pH, and effects of aquafeed ABC on the physiology and growth of fish

### 4.1 Gut pH in fish during digestion

To understand the impact of dietary acid–base capacity (ABC) on gut pH and digestion, it is essential to consider the spatio-temporal variations of gut pH in healthy fish, alongside the physiological principles and potential costs associated with the regulation of gastric and intestinal pH.

Numerous studies investigating the effects of diet ABC on the health and physiology of monogastric animals emphasize the implications for gut pH and gastric HCl secretion (8, 12, 25, 54–58). This focus is unsurprising, as the stomach serves as the primary digestive organ where ingested food experiences a substantial change in pH. Gastric acidity plays multiple roles during vertebrate digestion, contributing not only to protease activity but also to food disinfection and the solubilization of calcium and ferric ions (5). However, it is important to note that several fish taxa are secondarily agastric (59), such as the cyprinid family that makes a significant contribution to global finfish aquaculture production (1). In contrast, salmonids and many cultured marine fish species possess functional stomachs. Generally, fish exhibit a lower capacity for gastric acidification than mammals, with gastric pH values often approximating 4.0 during both inter-digestive periods (basal secretion) (60) and active digestion (6), with the exception of sharks, which may differ (61).

Similar to terrestrial vertebrates, hydrochloric acid is secreted into the stomach lumen of fish through microscopic pits located on the gastric epithelium, which are connected to gastric glands extending through the mucosal layer of the organ. The fundamental mechanism of gastric acid secretion has been conserved throughout vertebrate evolution, involving the H^+^/K^+^ ATPase (HK ATPase). This enzyme catalyzes the translocation of H^+^ ions from the cytoplasm to the gastric lumen via the apical plasma membrane of oxynticopeptic cells (59, 62).

The secretion of gastric HCl is under neurohormonal control (63), and it is also influenced the by the composition of the gastric chyme. For example, luminal calcium ions may activate the secretion (64), whereas low gastric pH levels exert negative feedback on secretion rates (60). These regulatory mechanisms are crucial in determining gastric pH during digestion. In any case, quantifying the total amount of gastric acid secreted during a digestion cycle, which is related to diet ABC, is difficult, since the rate of stomach emptying and the content of dissociable groups vary throughout digestion. The H^+^/K^+^ ATPase pumps protons into the stomach lumen with a stoichiometry of H^+^/ATP that varies between 1 and 2, depending on luminal pH (65). This area of research may have significant implications for the fish aquaculture industry, particularly if the gastric acidification of commercial diets constitutes a substantial portion of the animals’ energy expenditure.

Contrarily to the effects on gastric pH, the impact of diet ABC on intestinal pH is indirect. As previously discussed, the intestine of fish species with a functional stomach receives the gastric chyme rather than the original diet. The pertinent buffering variable in this context should be the base binding capacity (BBC) of the gastric chyme, which can be measured by titrating chyme with a basic compound from the initial acidic pH to the physiological pH in the fish intestine—typically ranging from 6.0 to 7.0 in the duodenum (which can be called BBC-6 and BBC-7), and between 8.0 and 9.0 in more distal sections (BBC-8 and BBC-9). The measurement of diet BBC has been previously measured by suspending the diet in water, and titrating the mix with a strong base until the endpoint (43). Since gastric chyme is a fluid with a high moisture, we propose mixing it with water at a low ratio, 1:1 (vol/vol), before measuring BBC. To date, no measurements of the BBC of gastric chyme in fish have been reported. However, several studies have examined the intestinal pH in fish fed diets with different ABC-3 and ABC-4 (13, 25, 58), but the potential correlation between diet ABC and gastric chyme BBC remains unexplored.

In fish, the intestinal alkalinity is primarily regulated by the secretion of bicarbonate ions (HCO₃^−^) into the intestinal lumen. The sources of intestinal bicarbonate include: (i) bile from the liver, (ii) enterocytes, and (iii) the exocrine pancreas, although distinguishing between the contributions of the latter two sources poses a challenge (66). This secretion process is governed by endocrine regulation (67). The regulation of intestinal bicarbonate fulfills at least two critical functions: (i) the production of calcium carbonate, which facilitates intestinal water absorption in marine fish, although this process is also active in freshwater species (66), and (ii) the neutralization of the acidic gastric chyme, resulting in mildly acidic or nearly neutral pH values (13, 25, 29, 53). Notably, the temporal fluctuations of intestinal pH during digestion are considerably smaller than those observed in the stomach, typically remaining within a range of less than 1 pH unit. As with gastric HCl secretion, the secretion of intestinal bicarbonate incurs an energetic cost (68). In experiments utilizing isolated intestines of *Opsanus beta* weighing up to 20 g, Taylor and Grosell (69) calculated that the proportional contribution of the intestine to the total SDA in fed animals was approximately 5%. It can be inferred that the contribution of bicarbonate secretion by enterocytes in that species is likely below 5%.

### 4.2 Aquafeed ABC and gut pH in fish

Tables 1, 2 summarize the main results about pH and ABC of experimental aquafeeds, and the reported effects on fish physiology were summarized in Table 4. The sequence of events shows how experimental objectives initially revolved around gastric pH, the measurement of dietary ABC, and the effect of plant ingredients, while more complex objectives related to fish specific dynamic action, the cost of gastric acid secretion, and the effects on growth have been addressed in recent years. The influence of dietary buffering properties on animal digestion and intestinal health has been extensively studied in domestic, laboratory, and some wild animals for various reasons, including cultured fish. Thus, our understanding of the effects of diet ABC on fish can be enhanced through comparisons with previous studies conducted on terrestrial vertebrates.

**Table 4:** Experiments on diet acid binding capacity (ABC), and gastric acid secretion in relation to fish digestive physiology.

Reference	Diet CP (%)	Additive	Diet ABC (mEq H^+^/kg)	Species	Growth time (d)	Physiological effects
Márquez et al. (7)	<5	0	48 (ABC-4)	*Sparus aurata*	–	Gastric pH inversely related to ABC
	>30	0	470 (ABC-4)			Minimum gastric pH does not depend on ABC
						Suboptimal activation of gastric proteases
Parma et al. (13)	47	0	750 (ABC-3)	*Dicentrarchus labrax*	64	Mild increase in pH of post. Intestine due to CaCO_3_
	47	0	530 (ABC-3)			
						Unaffected SGR and feed conversion rate
	47	3% CaCO_3_	750 (ABC-3)			
						Intestinal ratio Firmicutes:proteobacteria was lower in diets with a higher ABC
Goodrich et al. (12)	nd	0	600 (ABC-3.5)	*Lates calcarifer*	28	Decreased total SDA in fish fed the HCl diet
	nd	1.65% HCl	30 (ABC-3.5)			Gastric pH inversely related to ABC till hour 12
	nd	10.6% Na HEPES	1,040 (ABC-3.5)			
						Lowered food conversion in fish fed the HCl diet at day 14, but not at day 28.
Goodrich et al. (58)	nd	2.7% CaCl_2_ 2H_2_O	228 (ABC-3.5)	*Oncorhynchus mykiss*	21	Total SDA increased by 11% due to CaCO_3_
						Highest blood HCO_3_^−^ in fish fed Ca_3_(PO_4_)_2_ or CaCO_3_
	nd	1.9% Ca_3_(PO_4_)_2_	320 (ABC-3.5)			
						Highest growth for Ca_3_(PO_4_)_2_ diet, lowest for CaCO_3_ diet
	nd	1.8% CaCO_3_	550 (ABC-3.5)			
Moffatt et al. (25)	50	0.125–0.25% OM	941 (ABC-3)	*Oreochromis niloticus*	56	Higher gastric pH, and faster evacuation in OM fish
						SDA quantity and duration reduced in OM fish
						Decreased SGR in OM fish
Ciavoni et al. (14)	36	0	212 (ABC-3)	*Oncorhynchus mykiss*	42	Decreased growth rate in fish fed the CaCO_3_ diet
	35	3% CaCO_3_	328 (ABC-3)			
						Increased food conversion in fish fed the CaCO_3_ diet
	57.5	0	284 (ABC-3)			
	55.5	3% CaCO_3_	406 (ABC-3)			
						Mild increase in intestinal pH due to CaCO_3_
						Low ash and P digestibilities in fish fed the CaCO_3_ diet
Ruiz et al. (99)	36	0	212 (ABC-3)	*Oncorhynchus mykiss*		Intestinal Desulfobacterota decrease with CaCO_3_ diet
	35	3% CaCO_3_	328 (ABC-3)			
						The genus *Geobacillus* was present in the anterior intestine only in fish fed the high protein, no CaCO_3_ diet.
	57.5	0	284 (ABC-3)			
	55.5	3% CaCO_3_	406 (ABC-3)			
						*Geobacillus* was positively correlated with ADC of Mg.

CP, crude protein; OM, omeprazole; SDA, specific dynamic action; ADC, apparent digestibility coefficient.

Diet ABC can significantly impact gastric pH and the timing of gastric pH changes during digestion, in coordination with physiological regulation. For instance, the *ad libitum* ingestion of a commercial-type diet (ABC = 470 mEq/kg) caused gastric pH to decrease from 6.5 to 4.8 within the first 4 h of digestion in *Sparus aurata* weighing 20 g. Conversely, a diet with a very low ABC (ABC = 47 mEq/kg) led to a decrease in gastric pH from 6.5 to 4.6 in just 30 min, a value that was maintained for at least 1.5 additional hours (7). Although the same pH change was achieved more rapidly with the low ABC diet, the minimum gastric pH remained unchanged, likely due to physiological regulation. Similar patterns of gastric pH change have been observed when dietary pH values were comparable and diet ABCs were different (13, 14). When a low ABC diet was paired with a significantly lower dietary pH (as seen with dietary acidification), gastric pH can be substantially reduced for extended periods (12). These effects are expected to depend on fish species and life stage, but these topics remain experimentally unexplored. For example, the minimal gastric pH during digestion in the fish species *Sparus aurata* is above 5.0 in animals of 0.4–0.8 g, close to 4.5 in animals of 8 or 20 g, and can reach a value below 4.0 in juvenile animals weighing 150 g (7, 53, 70). The decrease in minimal gastric pH with fish size have been reported in other species, but the particular shape of the decreasing function is species-specific (71). These facts suggest that the practical endpoint to determine diet ABC should be selected in keeping with the fish species and animal size. Thus, for a given diet, the amount of gastric acid secreted per unit of mass of ingested diet probably increases with fish size as the minimal gastric pH during digestion decreases. On the other hand, the amount acid secreted into the stomach depends also on the potential relationship between fish size and the inclusion rate of vegetable ingredients.

In comparison with terrestrial monogastric animals, a positive correlation between gastric pH and diet ABC has been reported in piglets, alongside a gastric pH drop in the presence of dietary acidifiers (8). However, these authors did not differentiate between the effects of diet pH and diet ABC. Additionally, Ali and Evans (72) established a direct relationship between dietary buffering capacity and gastric pH in rats. The experiment implied the manipulation of dietary ratios of H₂PO₃^−^ to HPO₃^2−^, while maintaining phosphorus content.

Regarding the intestinal pH, Ciavoni et al. (14) supplemented a diet for rainbow trout with 3% CaCO_3_, such an inclusion increased diet ABC-3 by 120 mEq of H^+^ per kg in relation to the control diet, and led to a 0.2 pH unit increase in the pH of distal intestine. The alkalinization of distal intestine could also have resulted from the interactions between dietary calcium and dietary phosphate. For instance, in rats, calcium ions can partially interact with phosphate anions to form insoluble salts in the intestine, which are subsequently transported without being absorbed, ultimately reaching the lower intestine and cecum, where they help buffer intestinal pH—particularly under conditions of carbohydrate fermentation that produce low pH and compromise the barrier function (73–75). Similar interactions involving dietary calcium, phosphate, and fermentable carbohydrates (76), as well as the buffering properties of intestinal digesta, may occur in farmed fish. For example, Parma et al. (13) observed an increase in hindgut pH in *Dicentrarchus labrax* when fish were fed a plant-based diet supplemented with 3% CaCO₃, potentially related to the “phosphorus shuttle” mechanism (defined as the transportation of insoluble salts of P from the anterior to the posterior intestine) studied by Schepens et al. (75) in the rat intestine.

### 4.3 Aquafeed ABC and specific dynamic action (SDA) in fish

The specific dynamic action can be briefly defined as the energetic cost of the digestion and assimilation of a meal, that implies a postprandial rise in the metabolic rate of the animal (77). The effect of diet ABC, and/or of the gastric function workload, on the SDA of fishes and snakes (two groups of ectothermic monogastric animals) has been investigated by several authors, and the main results have been compiled in Table 5. This line of research was carried out earlier on snakes, and very recently on fish species. Although there are differences in the physiology of snakes and fishes, for example, larger rations in snakes (but not so large in terms of dry weight), and higher postprandial gastric pH in fishes, there are also similarities, for example, the gastric HCl-secreting cell is the oxyntopeptic cell type in both animal groups (5, 78–80). Finally, the apparent similarities in experimental design, research objectives, and conclusions between both animal groups warrant a careful comparison.

**Table 5:** Summary of experiments on the effect of diet ABC and/or gastric function on the SDA of fish and snake species.

Article	Species/Temp.	Diet/Factor	Diet ABC-3.5 mmol H^+^/kg	Ration % BW	Minimal gastric pH	SDA length (day)	SDA	SDA unit
Fishes								
Goodrich et al. (12)	*Lates calcarifer*	Pellets, HCl	30	3.5	<2.5	1.80	42.6	KJ/kg
	29.0 °C	Pellets, None	600	3.5	3.0	2.44	78.2	KJ/kg
		Pellets, HEPES	1,040	3.5	3.2	2.63	86.1	KJ/kg
Goodrich et al. (58)	*Oncorhynchus mykiss*	Pellets, CaCl_2_	228	2.5	2.1	5.35	53.5	KJ/kg
	15.0 °C	Pellets, Ca_3_(PO_4_)_2_	320	2.5	2.9	5.40	54.5	KJ/kg
		Pellets, CaCO_3_	550	2.5	3.4	5.15	59.3	KJ/kg
Moffatt et al. (25)	*Oreochromis niloticus*	Pellets, None	780	2.0	1.5^a^	2.66	246	mg O_2_/kg
	26.0 °C	Pellets, OM (25 mg/kg)	780	2.0	4.5^a^	1.74	161	mg O_2_/kg
Ferreira et al. 2026 (9)	*Astyanax mexicanus*	Worms, HK pump wild phenotype	66	5.0		0.64	21.5	mmol O_2_/kg
	22.3 °C	Worms, HK pump knockout	66	5.0		0.57	16.8	mmol O_2_/kg
Snakes								
Secor (83)	*Python molurus*	Rat, whole prey		25	1.5	8.00	528	KJ/kg
	30.0 °C	Rat, pureed prey		25		6.00	391	KJ/kg
		Rat, gastric bypass		25		6.00	175	KJ/kg
Andrade et al. (55)	*Boa constrictor*	Rodents, none		30			12160^b^	mL O_2_/kg (72 h)
	30.0 °C	Rodents, OM (22 mg/kg)		30			11810^b^	mL O_2_/kg (72 h)
Enok et al. (84)	*Python regius*	Mouse, none		18		>8.0	1,598	mL O_2_/kg (18 h)
	30.0 °C	Mouse, pyloric block		18		>8.0	623	mL O_2_/kg (18 h)
Norgaard et al. (56)	*Python regius*	Pureed mouse, 5.40 g bone/kg		15	3.2–2.2	>8.0	7,400	mL O_2_/kg (72 h)
	–	Pureed mouse, 20.5 g bone/kg		15		>8.0	7,130	mL O_2_/kg (72 h)
		Pureed mouse, 33.0 g bone/kg		15	2.0	>8.0	7,750	mL O_2_/kg (72 h)

a Gastric chyme pH after 12 h of digestion.

b Estimated from Figure 1 in Andrade et al. (55).

OM, omeprazole.

Among the studies about fish gastric function, Goodrich et al. (12) changed the ABC-3.5 of a commercial diet from 600 mEq kg^−1^ (control diet) to 30 mEq kg^−1^ by adding HCl. This modification resulted in a 45% reduction in total SDA in *Lates calcarifer*, with a decrease in postprandial oxygen consumption observed from the onset of digestion. Conversely, when dietary ABC-3.5 was increased from 600 (control diet) to 1,040 mEq kg^−1^ by adding the buffer HEPES, the total SDA increased modestly by 10%. Interestingly, the accumulated SDA over the first 12 h of digestion was nearly identical for both the control and HEPES-buffered diets, despite the buffered diet was associated with a significantly larger alkaline tide, indicative of a higher HCl secretion (81). In principle, the first comparison suggests that gastric workload contributes substantially to SDA, but the impact of HEPES diet on SDA does not strongly reinforce this conclusion. In another experiment, Goodrich et al. (58) found that more than doubling diet ABC (from 228 to 550 mmol H^+^ per kg), produced a 11% increase in the total SDA in the rainbow trout, pointing again to a modest contribution of gastric acid secretion to the cost of digestion in fish.

In a separate study by Moffat et al. (25), juvenile Nile tilapia was fed a diet containing omeprazole, a compound that inhibits gastric HCl secretion. It is interesting to note that the postprandial stomach of the Nile tilapia is as acidic as the stomach of *Boa* sp. or *Python* sp. snakes. This fact has been interpreted as a consequence of the particular diet of the Nile tilapia, which includes microalgae with hard cell walls (82). The investigation of Moffatt et al. (25) aimed to elucidate the effects of HCl secretion, and the implications of dietary ABC on fish growth. The authors confirm (from the titration curve of the diet, and the postprandial pattern of gastric pH) that the dose of omeprazole applied to fish inhibited the secretion of gastric acid by 90% at 12 h post-feeding. Moffat et al. (25) reported that dietary omeprazole led to a 35% reduction in SDA, and a corresponding 35% decrease in the duration of SDA. Thus, despite the inhibition of acid secretion, the SDA cost per unit of time remained relatively constant, suggesting a low contribution of HCl secretion to the overall SDA in Nile tilapia. In a more recent article by Ferreira et al. (9), the authors used a knock-out mutant of the freshwater fish *Astyanax mexicanus* that lacked a functional gastric proton pump, and compared mutant and wild-type phenotypes in terms of SDA after being fed a 5% bloodworm ration. Ferreira et al. (9) found that fish lacking a functional gastric proton pump exhibited a 11% reduction in the duration of the SDA, and a 22% reduction in the magnitude of the SDA. As in other experimental works, the authors stated that the reduction in SDA was not necessarily due only to the absence of gastric acid secretion. Indeed, the authors estimated the direct cost of gastric acidification (from diet ABC, gastric pH and the physiological cost of proton translocation), and obtained a contribution of about 2–6% of the whole SDA magnitude.

Snakes can consume large rations (relative to their body mass), and achieve gastric pH values as low as 1.5 to 2.0 for extended periods (83). However, in the experiments compiled in Table 5, snakes were always fed wet diets (whole or processed prey), whereas dry diets were used in the experiments with fish (barring (9)). This implies that rations in snake experiments were not so large in terms of dry weight. For example, if the moisture of prey is close to 70%, a ration of 18% of the snake body mass (% BW) in terms of prey wet weight, is equivalent to a ration of 5.4% BW in terms of prey dry weight. On the other hand, the diets supplied to fish species mentioned in Table 5 nearly always consisted of dry pellets, whose moisture is typically below 10%. A pellet ration of 2.5% would be equivalent to, at least, a 2.25% ration in a dry weight basis. Because of heavier rations, it is inferred that snake meals will be associated with a higher SDA, and most probably with a higher amount of secreted gastric HCl. Therefore, a higher ration does not necessarily imply a higher proportional contribution of the cost of HCl secretion to SDA, this proportion should be evaluated in each case.

In a pioneering work, Secor (83) evaluated the SDA associated with a pureed meal infused into the esophagus of the ophidian *Python molurus* versus the same meal infused into the proximal intestine, and observed a 55% reduction in SDA when the gastric function was bypassed. The energy difference was attributed to a variety of physiological processes such as gastric motility, enzyme synthesis, and HCl secretion, but the authors stressed the potential costs of HCl secretion associated with the meal’s buffering properties (not measured). Afterwards, in an experiment with the snake *Python regius*, Enok et al. (84) found that the contribution of gastric digestion to SDA was close to one-third at most, thus confirming a substantial energy expenditure due to the gastric function. On the other hand, Andrade et al. (55) reported nearly no difference in total SDA of *Boa constrictor* fed a ration comprising 30% of body wet weight, irrespective of the previous treatment of snakes’ stomach with omeprazole, a specific inhibitor of HCl secretion. Andrade et al. (55) did not measure gastric pH, but the authors confirm that the digestive treatment with omeprazole completely abolished the postprandial blood alkalinization (a direct consequence of the secretion of gastric acid), thus indicating a large inhibition rate of the secretion of acid. Additionally, Nørgaard et al. (56) manipulated the ABC of the food (by adding bone) while maintaining a nearly constant protein content, finding that a five-fold increase in food ABC-2 had no effect on SDA during the first 3 days of digestion (when HCl secretion is most active), and a total SDA increase of 23%, what supports a low cost of HCl secretion.

In summary, the experimental works support that gastric digestion significantly contributes to the specific dynamic action (SDA) in fish and snakes, but the impacts of diet ABC and HCl secretion are low or remain controversial. In experiments with omeprazole, more research is needed to understand the pharmacology of this compound when supplied to snakes and fishes at different temperatures, which complicates the comparison between both animal groups when subjected to the same dose of this inhibitor. In any event, assessing the energetic cost of HCl secretion through SDA data is challenging, since SDA can stem from multiple concurrent processes, including: (i) chyme alkalinization via bicarbonate secretion in the duodenum a few hours after feed ingestion (24, 85); (ii) active vasoconstriction in arterial circulation (86); (iii) excretion of the concurrent alkaline tide through ion transporters located in the gills and intestine (87, 88); (iv) mechanical contractions in the stomach (77).

### 4.4 Aquafeed ABC, growth rate, and phosphorus digestibility in fish

If diet ABC influences the costs of food digestion in fish, then it is reasonable to expect an effect on growth rates, a critical consideration for aquaculture species. Recent studies have investigated this relationship. Goodrich et al. (12) conducted a 28-day growth trial with juvenile *Lates calcarifer*, comparing a low ABC diet (supplemented with HCl) to a control diet. Fish consuming the acidified diet experienced a 12% reduction in feed conversion ratio (FCR) during the initial 14 days, along with a non-significant 7.4% increase in specific growth rate (SGR) during the same period. However, no differences in FCR or SGR were observed between diets during the final 2 weeks of the experiment. Given that SDA can account for 10–30% of the energy budget in fish (89, 90), a 45% reduction in SDA could reasonably lead to an 8% improvement in SGR, as noted in the first 14 days. The disappearance of this effect during the last 2 weeks warrants further investigation. In a separate experiment, Goodrich et al. (58) supplemented a commercial diet with various calcium salts—CaCl₂, Ca₃(PO₄)₂, or CaCO₃—to achieve ABC-3.5 values of 228, 320, and 550 mEq HCl kg^−1^, respectively. When these diets were supplied to juvenile *Oncorhynchus mykiss*, the highest SGR was associated with the Ca₃(PO₄)₂ diet, which had an ABC-3.5 that was 40% higher than that of the CaCl₂ diet. In contrast, fish fed the CaCO₃ diet exhibited the lowest SGR, showing a 12% reduction compared to those fed the CaCl₂ diet. The authors suggested that the enhanced availability of phosphorus in the Ca₃(PO₄)₂ diet might have interacted with the effects of ABC-3.5.

In another study involving *O. mykiss*, Ciavoni et al. (14) formulated four diets based on two factors: protein-to-energy ratio (16 or 26.4 mg kJ^−1^) and CaCO₃ supplementation (0 or 3%). The inclusion of dietary CaCO₃ increased ABC-3 by approximately 120 mEq H^+^ per kg of food, which decreased growth rates without affecting feed intake. This dietary change also resulted in reduced ash and phosphorus digestibility, while protein and energy digestibility remained unchanged. Similarly, Parma et al. (13) found that adding 3% CaCO₃ to a low-fishmeal diet for the marine species *Dicentrarchus labrax* led to a nearly significant 5% reduction in SGR, although phosphorus digestibility was not measured in this study.

This set of experiments indicates that the selection of buffering supplements to manipulate diet ABC is important. There is substantial evidence demonstrating the negative interaction between dietary calcium and phosphorus digestibility in both fish (91) and terrestrial monogastrics (92, 93). However, this dysergistic interaction between calcium and phosphate ions shows a complex dependency on gastric acidity. The phosphorus contained in certain salts such as di-Ca/Mg phosphate salts, tri-Ca/Mg phosphate salts, or bone-P salts (contained in fishmeal and animal protein ingredients), can be partially solubilized in the acidic environment of the stomach, and subsequently absorbed by the intestine of fish, otherwise the P of those sources remains nutritionally unavailable (94). On the other hand, when the diet contains the water-insoluble salt CaCO_3_, a low gastric pH solubilizes part of the calcium ions in the aqueous phase of the gastric chyme. After chyme evacuation into the duodenum, the solubilized Ca ions can precipitate, together with the solubilized phosphate ions, in the intestinal environment, diminishing the absorption of phosphate (95) and, in general, phosphorus digestibility (91). The extent of this digestibility reduction depends on the dietary ratio Ca:P, the higher the Ca:P ratio, the lower the P digestibility (95). Consequently, it is possible to hypothesize that, in the absence of CaCO_3_, a low diet ABC can promote gastric acidity and P digestibility. However, if diet formulation includes CaCO_3_, on the one hand, this compound increases diet ABC by itself (14), although this change in ABC not necessarily affects the minimum gastric pH during digestion (13). On the other hand, if diet ABC has been lowered despite containing CaCO_3_ (i.e., by including plant ingredients with a low ABC), the effect of a reduced ABC on P digestibility cannot be easily inferred, since it is expected to depend on the fractional increases of calcium and phosphate in the gastric chyme. These reasonings apply to the work conducted by Goodrich et al. (58), consisting in supplementing a commercial diet with different Ca salts. This experiment resulted in a higher SGR in fish fed the diet enriched in P, as Ca₃(PO₄)₂, not in fish fed the diet with the lower ABC (CaCl_2_ diet). Thus, the effects of diet ABC and P availability were most probably intermingled.

Consequently, interpreting the effects of dietary CaCO₃ on fish growth as solely attributable to its buffering properties is challenging. Since the requirement of calcium can be covered by waterborne calcium in the majority of fish species (96), it seems preferable avoiding the dietary inclusion of CaCO_3_ whenever possible. Similarly, when phosphate salts are utilized as dietary buffering compounds, disentangling the effects of ABC from the effects of additional phosphorus becomes difficult. As a consequence, the effects of different Ca salts reported by Goodrich et al. (58), or the effects of CaCO_3_ reported by Ciavoni et al. (14) on the growth rate of juvenile fish are probably related to the chemical interaction between calcium and phosphate ions. Therefore, to study the specific effects of diet ABC on fish growth, it would be more beneficial to manipulate ABC using salts devoid of calcium and phosphorus. On the other hand, the effect of adding HCl to the diet (12) can be more clearly attributed to a change in diet ABC, and it suggests a temporal effect of diet ABC on fish growth that warrants further investigation.

### 4.5 Aquafeed ABC and intestinal microbiota of fish

While the relationships between diet ABC, gastric pH and gut microbiota have been extensively studied in terrestrial monogastrics like pigs (8, 54) and chickens (57, 97, 98), this issue remains largely unexplored in cultured fish. Nevertheless, Parma et al. (13) published data on the hindgut microbiota of the marine fish *Dicentrarchus labrax* fed plant-based diets with varying CaCO₃ or fishmeal contents. Their study did not reveal strong differences in the intestinal microbiota, nor did it report data on survival rates. However, they observed a lower ratio of Firmicutes to Proteobacteria in fish fed diets containing CaCO₃, which was associated with a higher pH in the hindgut. In addition, Ruiz et al. (99) have recently investigated the effects of dietary protein-to-energy ratio, and the inclusion of CaCO_3_, on the gut microbiota of *Oncorhynchus mykiss*. The results of Ruiz et al. (99) support a reduction in the abundance of bacteria of the phylum Desulfobacterota in the proximal intestine of fish fed diets with CaCO_3_. The authors reasonably explained that result as a consequence of the increase in gastrointestinal pH promoted by dietary CaCO_3_ (previously reported by Ciavoni et al. (14) in the same species fed the same diets). Similarly, Ruiz et al. (99) also found that the abundance of the bacterial genus *Geobacillus* was increased in fish fed diets with a high protein-to-energy ratio, but only in the absence of CaCO_3_ which, again, can be due to the intestinal alkalinization promoted by the high ABC of CaCO_3_. Interestingly, the abundance of *Geobacillus*, a genus known to produce phytases (100), was positively correlated with the digestibility of Mg in the work by Ruiz et al. (99). In general, the scarce investigations on the effect of diet ABC on the microbiota of gut fish do not report any pathogenic effects. In contrast, diet ABC is recognized as a critical determinant of intestinal health in weaning piglets and chickens. The acidification of the gastric lumen in piglets relies on lactose fermentation by lactic acid bacteria. Thus, the transition from a lactose-rich lactation diet to a solid diet (with higher ABC and no lactose) during weaning leads to an increase in gastric pH. This increase can promote the growth of pathogenic microorganisms and can cause diarrhea (8, 54). In chickens, diet ABC can also trigger intestinal diseases (57). The main issue in chickens seems to stem from the dietary calcium supplied as limestone, primarily composed of CaCO₃. The high ABC of limestone results in: (i) an increase in gastric pH that varies with the age of the bird (97), potentially impairing the activity of gastric proteases and exogenous phytases (98), thus increasing nutrient availability for fermentation in the hindgut, and (ii) enhanced environmental conditions for the growth of *Clostridium perfringens* in the hindgut, which can cause necrotic enteritis (57, 98). Therefore, while dietary ABC may influence the intestinal microbiota in fish, especially in the hindgut, no clear associations with intestinal diseases or survival rates, similar to those observed in terrestrial monogastric vertebrates, have been reported in fish to date. This lack of strong effects of diet ABC on gut health in fish seems to imply an important asymmetry between livestock and fishes. It can also be a consequence of the immaturity of the research about diet ABC and gut microbiota in cultured fish. Lastly, since Parma et al. (13) and Ruiz et al. (99) manipulated diet ABC solely by including CaCO_3_, there is risk of confounding the effects of diet ABC with the more specific effects of CaCO_3_, such as simultaneous changes in dietary calcium and phosphorus availabilities. Thus, more robust results can be expected as new experiments testing the effect of diet ABC manipulated in different ways are published in the future.

## 5 Conclusion

Over the past decade, research findings have highlighted the significant impact of dietary buffering properties on the digestive physiology of cultured fish, in line with observations in other farmed animals. Increasing dietary ABC has been shown to raise gastric pH, influence specific dynamic action (SDA), and likely enhance gastric acid secretion in fish. However, the relationships between these effects and fish growth rates or the energy costs associated with gastric acid secretion warrant further investigation. If some mineral salts such as calcium carbonate, or tricalcium phosphates, are used to manipulate diet ABC, it is essential to take into account their intrinsic effects on the availability phosphorus when interpreting fish growth rates.

Several research directions can be proposed for future experiments related to the effects of diet ABC on farmed fish. In the first place, it is necessary to reach a consensus among researchers to design a standard method to measure the ABC of dietary ingredients and aquafeeds, so that variables like diet-to-water ratio, pH stabilization time, particle size or pH endpoints are standardized. Secondly, more research in necessary to assess the cost of gastric acid secretion in fish species. In this way, experiments with different inhibitors of the secretory pathway, or with knockout models of fish are encouraged. Thirdly, Since the original diet does not enter the intestine of fish species with a stomach, studying the buffering properties of gastric chyme should be researched by the application of additional concepts and experimental designs. Thus, it is proposed conducting more research on the base binding capacity of gastric chyme in fish, its correlation with dietary acid ABC, if any, and the subsequent effects on intestinal pH. Fourthly, another important theme which is not well studied in fish revolves around the effects of diet ABC on the intestinal microbiota. Some interesting objectives are the relationship between dietary non-starch polysaccharides (NSPs, diet ABC and the composition of intestinal microbiota, or the interactions between diet ABC and probiotics such as lactic acid bacteria). Another interesting topic poorly studied in fish is the interaction between calcium and phosphate ions in relation to diet ABC and intestinal pH. Intestinal digesta should be investigated in relation to pH and buffering properties, particularly in relation to dietary non-starch polysaccharides (NSPs), carbohydrate fermentation, and dysbiotic conditions in the distal intestine of fish. Finally, it is proposed to advance in the gastric physiology of larval fish related to diet ABC. On the one hand, the ABC of micro-diets for larval fish remain unexplored. On the other hand, the effects of diet ABC on gastric pH of larvae can be particularly important during the onset of the gastric function, when the secretion of HCl is not well established. The research with larvae is technically challenging, since the accurate measurement of gastric pH in such a small stomach is difficult. Nevertheless, this problem could be circumvented by the inclusion of pH indicators in the diet.
